# The True Colours Remote Symptom Monitoring System: A Decade of Evolution

**DOI:** 10.2196/15188

**Published:** 2020-01-15

**Authors:** Sarah M Goodday, Lauren Atkinson, Guy Goodwin, Kate Saunders, Matthew South, Clare Mackay, Mike Denis, Chris Hinds, Mary-Jane Attenburrow, Jim Davies, James Welch, William Stevens, Karen Mansfield, Juulia Suvilehto, John Geddes

**Affiliations:** 1 Department of Psychiatry University of Oxford Oxford United Kingdom; 2 4YouandMe Seattle, WA United States; 3 Oxford Center for Human Brain Activity University of Oxford Oxford United Kingdom; 4 Oxford Health NHS Foundation Trust Oxford United Kingdom; 5 Big Data Institute University of Oxford Oxford United Kingdom; 6 Department of Computer Science University of Oxford Oxford United Kingdom; 7 NIHR Oxford Biomedical Research Centre, University of Oxford Oxford United Kingdom

**Keywords:** symptom assessment, signs and symptoms, digital health, ecological momentary assessment, mood disorders

## Abstract

The True Colours remote mood monitoring system was developed over a decade ago by researchers, psychiatrists, and software engineers at the University of Oxford to allow patients to report on a range of symptoms via text messages, Web interfaces, or mobile phone apps. The system has evolved to encompass a wide range of measures, including psychiatric symptoms, quality of life, and medication. Patients are prompted to provide data according to an agreed personal schedule: weekly, daily, or at specific times during the day. The system has been applied across a number of different populations, for the reporting of mood, anxiety, substance use, eating and personality disorders, psychosis, self-harm, and inflammatory bowel disease, and it has shown good compliance. Over the past decade, there have been over 36,000 registered True Colours patients and participants in the United Kingdom, with more than 20 deployments of the system supporting clinical service and research delivery. The system has been adopted for routine clinical care in mental health services, supporting more than 3000 adult patients in secondary care, and 27,263 adolescent patients are currently registered within Oxfordshire and Buckinghamshire. The system has also proven to be an invaluable scientific resource as a platform for research into mood instability and as an electronic outcome measure in randomized controlled trials. This paper aimed to report on the existing applications of the system, setting out lessons learned, and to discuss the implications for tailored symptom monitoring, as well as the barriers to implementation at a larger scale.

## Introduction

The advancement of digital technology will gradually continue to shape how we measure, monitor, and manage health. A wide range of digital symptom monitoring tools exist, but there is a lack of evidence regarding their effectiveness in a health care context, particularly in the area of mental health. Such evidence will arise only from studies involving significant usage, conducted in close partnership with clinicians, patients, and managers. For example, digital tools for patient-reported outcome measures (PROMs) are becoming standard practice in randomized controlled trials (RCTs) in many areas [1], and meta-analyses [2,3] have confirmed their equivalence with paper-based approaches.

True Colours is a digital tool, developed over a decade ago by psychiatrists, software engineers, and researchers at the University of Oxford, which has achieved significant usage. The initial version was used for remote monitoring of mood disorders, allowing patients and their clinicians to record and review symptom change. The recognized need to capture and monitor higher frequency phenotype information, particularly for conditions such as bipolar disorder (BD), is not new. Hard copy symptom monitoring diaries have been used for decades. However, these are limited by practicality issues.

The True Colours system has many advantages over paper-based approaches toward the capturing of detailed, timed phenotype information, including the following: the ability to prompt for contemporaneous input, the automatic calculation of summary scores, the visualization of changes over time, and the provision of real time, as well as historical data to support clinical review, assessment, and early intervention. From a research perspective, the tool has additional advantages: eliminating errors in the transcription of information from paper forms, supporting a higher frequency of prompted, directed phenotyping, and reducing the recall bias associated with the recording of symptoms. Subsequent versions of the tool have added new functionality for data entry, patient or cohort management, and research delivery.

The system has been applied across several patient, participant, and high-risk populations, being used across 21 unique research and clinical service settings in the Oxfordshire and Buckinghamshire regions in the United Kingdom. Over the past decade, there have been over 36,000 registered True Colours participants from whom over 1.4 million questionnaire responses have been collected. Several feasibility studies and clinical service applications support the potential of True Colours as a larger scale symptom monitoring system, an electronic PROM, and a tool for digital phenotyping. This paper aimed to describe the evolution of the tool, its applications, and achievements and to discuss the potential for future wider application and integration.

### Research Applications

**Figure 1:**
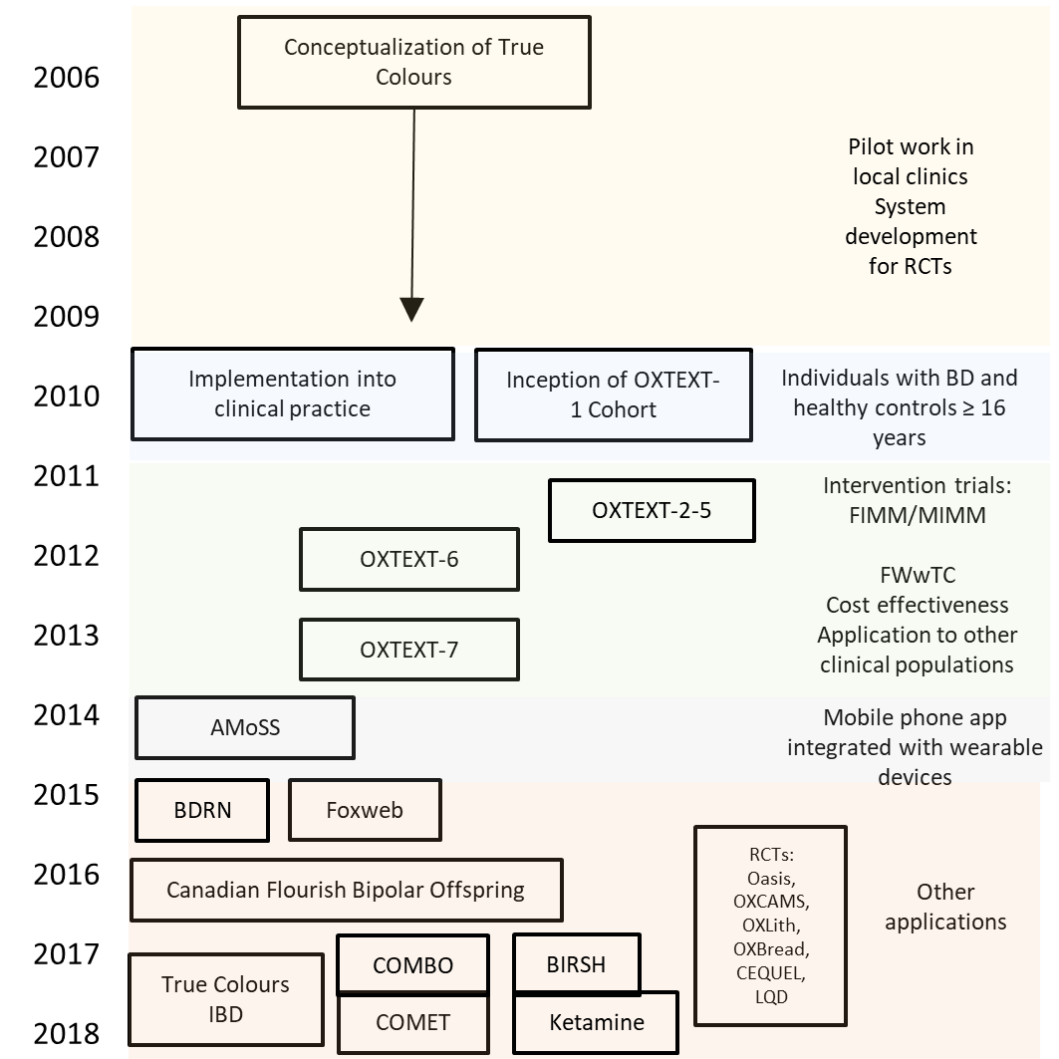
Evolution and applications of True Colours. AMoSS: Automated Monitoring of Symptom Severity Study; BD: bipolar disorder; BDRN: Bipolar Disorder Research Network; CEQUEL: Comparative evaluation of quetiapine plus lamotrigine; COMBO: Collaborative Care Model for Bipolar Disorder; FIMM: Facilitated Integrated Mood Management; FWwTC: Feeling Well with True Colours; IBD: inflammatory bowel disease; LQD: lithium versus quetiapine augmentation for treatment resistant depression; MIMM: Manualized Integrated Mood Management; OxBREaD: Oxford Brain Body Research into Eating Disorders; OxCAMS: Oxford Study of Calcium Channel Antagonism, Cognition, Mood instability and Sleep; OxLith: Oxford Lithium Trial; RCT: randomized controlled trial.

The True Colours system was originally designed to monitor mood symptoms in adult patients with BD, attending the BD Research Clinic at the Department of Psychiatry at the University of Oxford, and it was designed for use in clinical trials, evolving from the Oxford University Symptom Monitoring System [4,5]. The original version of the system involved automated weekly prompts, delivered by text message or email (chosen by preference), for patients to complete self-reported measures of symptoms, including depression (16-item Quick Inventory of depressive symptoms) [6] and mania (5-item Altman Self Rating Mania Scale) [7], and other measures, such as anxiety (Generalized Anxiety Disorder Scale–7) [8], quality of life (EQ-5D) [9], and lifestyle behaviors. The system has expanded to include a wide range of symptoms from validated scales and bespoke measures tailored to specific research projects. As part of the True Colours platform, total symptom scores were presented graphically via a secure website and made available to patients, participants, and clinicians upon request. Over the past decade, the use of True Colours has expanded to several different research cohorts and patient populations (Figure 1).

#### The OXTEXT Program

Earlier work involving the University of Oxford Symptom Monitoring System established that technology-assisted symptom monitoring was acceptable to patients over a period of 36 weeks with 75% compliance [4], meaning that, on average, patients reported symptoms in response to prompts 75% of the time over follow-up. The OXTEXT program was dedicated to developing and validating the True Colours remote symptom monitoring system for patients, at a larger scale. Across several projects, the program revised the software after in-depth patient consultation, established a cohort of well-characterized patients with BD by using the improved system, determined the potential cost-effectiveness of this remote capture tool in clinical service, and tested remote mood monitoring as a potential intervention via RCTs (OXTEXT research studies 1-6). Several publications resulted from these studies, largely from the OXTEXT-1 cohort comprising up to 367 patients (≥16 years of age) from Oxfordshire, with a Diagnostic and Statistical Manual of Mental Disorders-IV diagnosis of BD (BDI, BDII, or BD-not otherwise specified), with some patients completing up to 81 months of continuous weekly mood measures. Compliance and acceptability of the True Colours system in the OXTEXT-1 cohort were excellent, with low attrition (<2%) and a median of less than 8% of weeks of missing data that did not differ by key sociodemographic factors or by mood score [10]. This pilot work has demonstrated support for the feasibility of True Colours as a remote mood monitoring system in patients with mood disorders [4,10], and it has lent important insights into detectable mood instability that differentiates clinical course [5] and other BD patient characteristics, including cognitive functioning [10,11].

The OXTEXT-2 study [12] assessed participant’s compliance with monitoring, their mental health resource use (including hospitalizations and face-to-face and phone contact with mental health staff), and service and medication costs, before and during their first 12 months of engagement with True Colours. Compliance with monitoring was high, with a median response completion rate of 92% for both Web-based and SMS symptom reporting and all patients continuing to report during the duration of the study. The introduction of True Colours was thought likely to reduce service costs, but this was not supported in OXTEXT-2. In fact, when associated with enhanced specialist care, medication costs increased over the first year of monitoring. This illustrated that studies of any digital addition to care need to account for all possible confounders relating to mood monitoring and mental health service costs. OXTEXT-2 did not examine nonmental health service costs, and larger economic evaluations of the True Colours system are required and are being conducted.

The True Colours system was also utilized as part of a psycho-education intervention for 121 patients with BD in an RCT (OXTEXT-6) [13]. The Facilitated Integrated Mood Management (FIMM) [14] study condition involved True Colours mood monitoring, a psycho-education manual, and individual sessions with a facilitator. This was compared with Manualized Integrated Mood Management, which only involved the psycho-education manual. Patients in the FIMM arm showed better knowledge of BD, and greater BD knowledge was associated with a high number of months in remission over 1-year follow-up [13]. Of note, True Colours in isolation is not intended as an intervention, but it may improve symptoms via insight into patients about their symptoms and closer, more accurate monitoring by clinicians, which will require further study.

The OXTEXT-7 study commenced in 2013 involving a trial rolled out to all 11 community mental health treatment services across Oxfordshire and Buckinghamshire titled as Feeling Well with True Colours (FWwTC). The goal of FWwTC was to offer patients a self-monitoring system that could allow care interventions to be tailored to the individual. Patients and clinicians create tailored symptom monitoring schedules on the basis of the type of symptom measure, frequency of prompts (weekly, daily, and several times a day), and reminder frequency. This study was a stepped-wedge, cluster randomized design. In this design, all services eventually implemented FWwTC, but the time at which they were trained to implement FWwTC was randomized to compare outcomes in treatment services before and after the introduction of FWwTC. The aim of this phase of OXTEXT was to apply True Colours to other patient populations (including those experiencing depression, anxiety, psychosis, alcohol and drug use, and BD) and test the feasibility and cost-effectiveness of such a tool in a larger scale secondary care setting. Experience from this trial is currently being synthesized, and it has proved heuristically useful [15], although uptake across clinical services was a challenge, illustrating the considerable barriers to innovation that persist in the National Health Service and other medical services.

### Digital Phenotyping Studies

Digital phenotyping is the individual-level high-resolution data capture enabled by digital devices. The promise in this data capture is its ability to collect passive or active information in a real-world setting unbound to clinical visits. This affords the opportunity to discover new trajectories of signs and symptoms of disease, resulting in refined phenotypes and better detection and management of illness. The Collaborative Network for Bipolar Research to Improve Outcomes (ConBrio) [16] was a translational research program aimed at bringing together basic and clinician scientists in mathematics, computational biology, cognitive neuroscience, and neuroimaging. Central to the ConBrio program is the use of True Colours complemented by other methods for deep and frequent mood phenotyping to accelerate understanding and treatment of BD. This program has supported several projects, such as the Automated Monitoring of Symptom Severity Study (AMoSS), the use of True Colours in several RCTs, for example, Oxford Study of Calcium Channel Antagonism, Cognition, Mood instability and Sleep (OxCaMS) and Oxford Lithium Trial (OxLith), and other large phenotyping studies from the BD Research Network (BDRN) [17].

#### Automated Monitoring of Symptom Severity Study

Taking advantage of the developments in digital technology and ubiquity of mobile phones, the AMoSS study introduced a mobile phone app, Mood Zoom, to facilitate a higher frequency of symptom monitoring and included wearable devices as measures of objective symptoms. The Mood Zoom questionnaire comprises mood state descriptor items that are rated on a scale from 1 to 7 [18], which could be completed several times a day. Mood Zoom was used alongside weekly True Colours mood monitoring to help understand, in greater detail, mood episodes and mood instability in patients with BD and borderline personality disorder, as well as healthy volunteers in a sample of 139 patients with 3 months of continuous data (as per protocol) but with over 12 months of continuous data (for those willing to continue). The introduction of a mobile phone app also enabled the collection of passive background data, such as number of texts or calls and geolocation [19], which could reflect proxies of behavior associated with BD and how they are associated with mood, an emerging area with promise for the identification of behavioral markers of impending BD-related episodes [20]. Quantitative [18,21-23] studies have supported the feasibility and acceptability of the use of the Mood Zoom app and True Colours for daily and weekly symptom monitoring in patients with BD, borderline personality disorder, and controls. Specifically, attrition was low in the AMoSS cohort, with only 1 subject withdrawing and 8 subjects being excluded because of providing data for less than 2 months. Median adherence for the Mood Zoom and weekly measures was greater than 80% and 85%, respectively, and it remained stable over the study follow-up [18]. A qualitative study of 20 subjects from the AMoSS cohort provided support for the fact that reporting on symptoms once daily was of no inconvenience, and it was felt that the system contributed to insights into personal symptoms and patterns [24]. Additional themes from this study highlight the importance of tailoring patient preferences into symptom reporting tools.

In recent studies, additional objective physiological measures, derived from Fitbit and wrist-worn accelerometers, were included, along with daily and weekly mood monitoring [22] as well as the proteus patch [21,25] that provides an estimate of heart rate. These studies have contributed insights into detectable variability of sleep patterns in patients with BD and borderline personality disorder, which map onto observable symptoms of low and irritable mood [21] and variability in mood [25]. The additional add-on of wearables offers an exciting line of inquiry into objective symptoms of illness-alleviating biases relating to subjective reporting of symptoms. This potentially supports downstream applications of True Colours, with the inclusion of additional devices for the measurement of objective symptoms, which will be important for deeper insights into early signs of disease.

#### Other Mood-Related Research Applications

The BDRN [17] adopted the True Colours system, engaging 815 research participants (815/4080, 19.97% of invited existing BDRN participants) with mood disorders [26]. BDRN participants with a diagnosis of BDII were more likely to register with True Colours. Approximately 78.2% (637/815) of registered participants completed 3 months of symptom reporting, approximately 51.1% (413/808) of the participants completed more than 1 year, and some participants continued mood monitoring for up to 3 years, demonstrating the feasibility of such a remote mood monitoring system at a larger scale.

An international application of True Colours is from the Canadian Flourish High-risk Offspring Study [27], recruiting young offspring of a parent with BD. The Flourish group has piloted the Web-based True Colours monitoring system in 50 high-risk offspring of a bipolar parent and 108 control offspring of psychiatrically well parents. Compliance was good over 30 days, with approximately 80% and greater than 90% of high-risk and control offspring completing daily ratings, respectively, and no difference in compliance between study groups. Daily mood scores significantly differentiated the high-risk from control offspring, and irregularity in weekly mood and anxiety scores was higher in high-risk offspring with remitted major mood disorders compared with those with no lifetime history of major mood disorders [27].

Additional studies from the University of Oxford have made use of the True Colours system to elucidate mood variability in BD, involving determining the different nonlinear time series processes of mood instability and analytic techniques for appropriately detecting it from high-frequency time series data [5,28], as well as its associations with mental imagery [29].

#### Application to Randomized Controlled Trials

RCTs of treatment efficacy in psychiatric disorders are expensive and lengthy, given the needed follow-up time for full Diagnostic and Statistical Manual of Mental Disorders threshold mood episodes to develop. Traditional endpoint assessments using paper and pencil questionnaires or clinician-rated diagnostic episodes also ignore clinically significant symptoms not meeting full diagnostic threshold between episodes [30] and cognitive dysfunction [31], which could be used to determine earlier and more proximal treatment effects. Several RCTs have used True Colours as both primary electronic outcome assessments and secondary higher frequency outcome measurements. For example, a 12-week double blind RCT (CEQUEL) [32] assessed combination therapy with quetiapine plus lamotrigine versus quetiapine monotherapy plus lamotrigine placebo on depressive symptoms in 266 patients (≥16 years) with BD, recruited across 27 different United Kingdom clinics. Another completed single blind RCT (OASIS) [33] of 3755 university students across the United Kingdom used True Colours to measure outcomes to determine the effectiveness of a Web-based cognitive behavioral therapy for insomnia and other psychiatric symptoms, including psychosis, mood, and anxiety.

Other mood-related applications of True Colours for outcome assessment in ongoing RCTs include the OxLith [34], aimed to compare lithium with placebo on mood instability in adult patients with BD; a trial assessing the clinical effectiveness and cost-effectiveness of lithium versus quetiapine augmentation for treatment-resistant depression [35]; and OxCaMS [36], which aims to assess the impact of a calcium channel blocker on cognition and brain activity in adults with mood instability. Finally, the Oxford Brain Body Research into Eating Disorders study [37] involves a pilot trial to assess the safety, acceptability, and feasibility of deep brain stimulation in patients diagnosed with severe eating disorders. Other funded large trials involving the True Colours system under development include the Pramipexole Therapy in Treatment Resistant Depression and Bipolar Depression (PAX-D and PAX-BD) [38,39].

#### Expansion to Other Populations

As the research and clinical utility of True Colours became evident, it naturally branched out to other populations and research contexts. The Cognition and Mood Evolution across Time study is aimed at measuring cognition and brain activity in healthy participants with various levels of mood instability—a useful application of True Colours, with the inclusion of daily mood monitoring and cognitive tasks [40].

The True Colours system has also been modified for community outpatients, with a diagnosis of psychosis using forensic psychiatric services (FOXWEB risk violence tool). This research application involved the development of a Web-based violence risk monitoring tool for psychosis, which provides visual feedback of patient scores to clinicians to guide risk assessment [41], and this is being further piloted in inpatients.

The Brief Interventions for Self-Harm (BIRSH) clinic [42] has piloted True Colours for self-harm prevention in patients (13-65 years) presenting to accident and emergency departments. The aim of this ongoing research and service evaluation application was to determine the effectiveness of a new clinical service incorporating remote symptom monitoring to reduce self-harm repetition and health service costs.

The True Colours inflammatory bowel disease (IBD) group has expanded the True Colours schedule to include daily measures of ulcerative colitis and Crohn’s disease symptoms, as well as fortnightly quality-of-life and other validated measures of disease activity. The initial aim of the True Colours IBD project was to develop and test the feasibility of a predictive index of IBD. A 6-month pilot in 66 patients supported the initial feasibility of this system, with 76% adherence rate for daily measures and 86% patient retention [43]. Further work has supported associations between daily IBD symptom measures and biological measures of disease activity [44], and this has facilitated the prediction of whether escalation of therapy or clinical investigation would be needed [45]. Qualitative findings from this work suggest that patients felt more in control and empowered by the True Colours IBD system [43].

### Clinical Service Applications

Several of the noted research applications have evolved into the use of True Colours for a purely patient monitoring and/or clinician monitoring tool, despite little infrastructure and resources to do so. As of January 10, 2019, almost 3000 patients with any psychiatric condition and more than 700 clinicians have registered with True Colours in adult community mental health treatment service clinics across Oxfordshire and Buckinghamshire. The uniqueness of this application of remote monitoring of symptoms is in the individualized approach. This enables patients to choose, in consultation with their health care professional, how they would like to self-monitor, directly aligning from qualitative work suggesting the preference of flexibility and personalization in a symptom monitoring tool [24]. The system has also been taken up by child and adolescent mental health services across the Oxfordshire region, with 27,263 registered users.

True Colours IBD is a prime showcase of what True Colours could evolve into—an integrated platform for individualized patient and clinician monitoring of symptoms and quality-of-life outcomes, with the potential to predict when more symptoms are expected and prevent unnecessary clinic visits. With further validation, the implications this model could have for reducing health care costs and burden on individuals are extensive. Since September 2019, there are currently more than 750 registered IBD patients, within the John Radcliffe Hospital in Oxfordshire, using True Colours as a monitoring tool. True Colours has also been applied as a patient-reported outcome monitoring tool in clinical service clinics, testing the effectiveness of Ketamine as a therapy for treatment-resistant depression [46] and for self-harm risk assessment as an extension to the ongoing pilot work conducted by BIRSH [42]. Finally, the Collaborative Care Model for BD is an ongoing project aimed at testing the feasibility of True Colours in a primary care setting to understand perspectives of the True Colours system from both patients and clinicians. This project also aims to engage different services (primary and secondary care clinicians) in the collaborative treatment of patients through the sharing of True Colours symptom ratings.

## Discussion

Over the past decade, True Colours has transformed from a simple text message prompt and reply system to a personalized Web-based symptom monitoring tool. This tool is now applied across a number of clinical populations and is integrated into several clinics as part of routine clinical care across the Oxfordshire and Buckinghamshire regions. A small team at the University of Oxford and the Big Data Institute has been supporting the continued use of True Colours and its application across a wide range of settings. Despite the relatively little resource that has been put into sustaining this system, its progress and scale, to date, are quite impressive, largely driven by small independent research grants.

The utility of True Colours as a research tool is unequivocal. The existing research involving this tool has contributed to considerable advancements in knowledge of mood instability and its correlates in mood and personality disorders, which would not have been possible with traditional aperiodic research or clinic assessments. The potential linkage of True Colours’ patient-reported data to electronic medical records data currently available within United Kingdom–Clinical Record Interactive Search—a national research platform comprising deidentified electronic patient medical records—could yield a rich source of high-frequency phenotyping information for future research. This data linkage could provide continuous measures of patient-reported symptoms occurring in real time, which could be mapped onto hospital visits and acute episodes of illness. This could afford the opportunity to fill in the gaps between clinic visits and determine early subsyndromal phases of illness that could reflect targets for prevention of episode recurrence or worsening of symptoms—a substantial scientific and clinical resource.

In 2017, there were 325,000 mobile health apps available internationally, including lifestyle interventions, symptoms trackers, and personal coaches [47]. A vast majority of these tools are not evidence based, and their ability to accurately measure symptoms or feasibly engage patients is largely unknown [48,49]. Only about 25% of digital health app users continue using the app after 10 uses [50], indicating challenges with low retention. Furthermore, with the rapid turnaround of digital health apps, it is difficult to rigorously test their effectiveness or implement into practice before they become obsolete [51]. Other symptom monitoring platforms include the Chrono-record [52], a computer-based symptom monitoring system, and the MONitoring treatment and pRediCtion BD episode system [53], an Android-based mobile phone objective and subjective symptom monitoring system designed for patients with BD. Patientslikeme [54] is a digital health platform in the United Kingdom, which involves a Web-based system that enables patients to track symptoms and view other members’ health information. The *Patientslikeme* platform currently has 600,000 registered users, and it is meant to produce data for research purposes and provide empowerment and community to patients to track their own symptoms. These tools are useful in unique ways, but these are yet to have any integration with clinical service. In addition, they are targeted toward specific conditions or the broad reporting of symptoms, some untethered to validated measures.

In an era where the digital health market is becoming increasingly saturated, careful integration of these tools within the health care system is crucial [55]. There is a need to develop digital remote monitoring tools that are evidence based [56], with infrastructure to support secure and sensitive personal information and enable the growth of the tool in tandem with rapidly developing digital technologies. Obvious barriers to this potential integration surround buy-in from health care providers, the potential to create inefficiencies, and data security concerns. This underscores the needed infrastructure for such a remote monitoring tool in clinical practice, with education for clinicians on its purpose and use, an electronic system with ease of access, and the flexibility and support to tailor the service to different patient populations and clinical care contexts. Uptake within clinical service will be a challenge and will require support from several participating parties.

What is unique about True Colours is the pilot work behind the tool’s feasibility across different patient populations, and its use alongside clinical judgement. Its evolution has been guided by several feasibility studies, clinical and software development expertise, and, most importantly, participant, patient, and clinician feedback. The concept of True Colours as an integrated clinical care model offers benefits to patients through the returning of simple, visually effective symptom summaries, empowering individuals to play an active role in their health, which alone could have a therapeutic effect, as seen in other areas of medicine, such as oncology [57,58]. For clinical practice, this tool could enable clinicians to have access to continuous health information from their patients unbound to clinic visits, providing PROMs at higher frequencies and lending insight into dynamic fluctuations in symptoms that cannot be captured by traditional health measurement systems by self-report measures recalling symptoms over long periods of time. In turn, this could support real-time assessment and management of chronic conditions while freeing up time and resources for the National Health Service.

## Abbreviations

AMoSS: Automated Monitoring of Symptom Severity Study
BD: bipolar disorder
BDRN: BD Research Network
BIRSH: Brief Interventions for Self-Harm
ConBrio: Collaborative Network for Bipolar Research to Improve Outcomes
FIMM: Facilitated Integrated Mood Management
FWwTC: Feeling Well with True Colours
IBD: inflammatory bowel disease
NIHR: National Institute for Health Research
OxCaMS: Oxford Study of Calcium Channel Antagonism, Cognition, Mood instability and Sleep
OxLith: Oxford Lithium Trial
PROM: patient-reported outcome measure
RCT: randomized controlled trial
